# Functional Poly(dihalopentadiene)s: Stereoselective Synthesis, Aggregation-Enhanced Emission and Sensitive Detection of Explosives

**DOI:** 10.3390/polym10080821

**Published:** 2018-07-25

**Authors:** Ting Han, Yun Zhang, Benzhao He, Jacky W. Y. Lam, Ben Zhong Tang

**Affiliations:** 1 HKUST-Shenzhen Research Institute, No. 9 Yuexing 1st RD, South Area, Hi-tech Park, Nanshan, Shenzhen 518057, China; thanac@ust.hk (T.H.); yzhangcl@connect.ust.hk (Y.Z.); hebenzhao@ust.hk (B.H.); 2 Department of Chemistry, The Hong Kong Branch of Chinese National Engineering Research Center for Tissue Restoration and Reconstruction, Institute for Advanced Study, Department of Chemical and Biological Engineering and Division of Life Science, The Hong Kong University of Science and Technology, Clear Water Bay, Kowloon, Hong Kong, China; 3 NSFC Center for Luminescence from Molecular Aggregates, SCUT-HKUST Joint Research Institute, State Key Laboratory of Luminescent Materials and Devices, South China University of Technology, Guangzhou 510640, China

**Keywords:** multicomponent polymerization, stereoselectivity, aggregation-enhanced emission, explosive detection, high refractive index

## Abstract

The development of polymeric materials with novel structures and unique properties and functionalities is of both academic and industrial significance. In this work, functional poly(dihalopentadiene)s were synthesized by boron trihalide-mediated multicomponent polymerization routes in a stereoselective manner. The polymerizations of tetraphenylethylene-containing diyne, BX_3_ (X = Cl, Br) and *p*-tolualdehyde proceed smoothly in dichloromethane under mild conditions to afford high molecular weight poly(dihalopentadiene)s with a predominant (*Z*,*Z*)-configuration in moderate to good yields. The reaction conditions and the boron trihalide used were found to have great effects on the stereochemistry of the resulting polymer structures. The obtained poly(1,5-dihalo-(*Z*,*Z*)-1,4-pentadiene)s possess high thermal stability and good film-forming ability. Their thin films show high refractive index of 1.9007–1.6462 in a wide wavelength region of 380–890 nm with low optical dispersion. The polymers are weakly emissive in dilute solutions but become highly emissive upon aggregated, demonstrating a unique phenomenon of aggregation-enhanced emission. Their nanoaggregates in aqueous media can serve as sensitive fluorescent chemosensors for the detection of explosives with a superamplification effect and a low detection limit.

## 1. Introduction

The construction of new functional polymers with unique structures and properties and potential high-tech applications is an enduring research topic in polymer science due to its great academic and industrial significance. Most of the commonly used functional polymers, such as polyolefins, polyesters and polyamides are constructed by the well-established homo- and two-component polymerizations [1,2]. However, these methods have become hard to meet the increasing demand on new polymeric materials with well-defined and complicated structures and multi-functionalities. Recently, multicomponent polymerizations (MCPs) derived from the fascinating multicomponent reactions (MCRs) [3,4,5,6,7,8,9] have received great attention and become a new research frontier in polymer science [10]. In MCPs, the reaction components are added into one reactor in one step or in a sequential/cascade manner without isolating the reactive intermediates [11,12]. From the synthetic point of view, MCPs generally have the merits of simple synthetic and isolation procedures, mild reaction condition, good functional-group tolerance, high efficiency, and free of waste formation. Furthermore, the simultaneous incorporation of three or more monomers with different functionalities in a one-pot MCP greatly enriches the structural diversity and properties of the resulting polymers [13,14,15,16].

The unique features of MCPs enable them to be effective tools for the preparation of functional polymers with complex and well-defined structures that are hard to be accessed by other polymerization approaches [17,18,19,20,21,22]. For example, a variety of heteroatom-rich polymers with multifunctionalities such as poly(*N*-sulfonylamidine)s [23,24,25], poly(*N*-sulfonylimidate)s [24,26,27], poly(iminocoumarin)s [28], poly(phosphorus amidine)s [29] and oxindole-containing poly(*N*-acylsulfonamide)s [30] have been facilely and efficiently synthesized through the Cu(I)-catalyzed one-pot MCPs of alkynes, sulfonyl azides and different nucleophiles. Sequence-controlled polymers have also been achieved by one-pot multi-step MCPs [25,31]. As some regio- and stereoselective MCRs have been developed and reported, regio- and stereoregular polymers are possible to be produced from the derived MCPs. For instance, inspired by the high regio- and stereoselectivity of MCRs of alkyne, carbonyl chloride, and secondary amines [32,33,34,35,36], our group have developed an efficient MCP of diyne, terephthaloyl chloride, and secondary aliphatic amines to synthesize functional poly(arylene enaminone)s with high content of *E*-configuration of up to 93% [37]. When the secondary amines were replaced by primary amines, regio- and stereoregular poly(enaminone)s with sole *Z*-configuration were generated in nearly quantitative yields [38]. The high regio- and stereoregularity of the polymer structure were attributed to the formation of strong intramolecular hydrogen bonds between the C=O and N–H groups in the resulting hydroamination products, which not only stablized the *Z*-configuration by blocking the *E*/*Z* isomerization but also inhibited the enamine-imine tautomerization process. Despite of the rapid progress of MCPs in the past decade, examples of powerful MCPs toward regio-/stereoregular functional polymers are still limited possibly due to the lack of efficient MCRs with high chemo-/regio-/stereoselectivity.

**Scheme 1:**
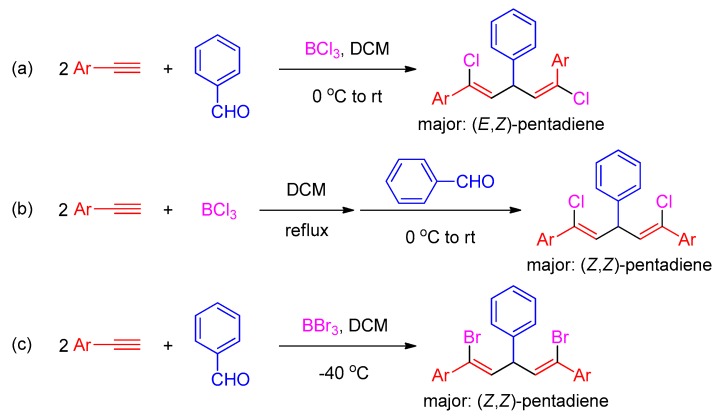
Boron trihalide-mediated alkyne-aldehyde coupling reactions toward 1,5-dihalo-1,4-pentadienes with different stereochemistry: (**a**) BCl_3_-mediated alkyne-aldehyde reaction toward (*E*,*Z*)-1,5-dichloro-1,4-pentadiene; (**b**) BCl_3_- and (**c**) BBr_3_-mediated alkyne-aldehyde reaction toward (*Z*,*Z*)-1,5-dichloro-1,4-pentadiene.

**Scheme 2:**
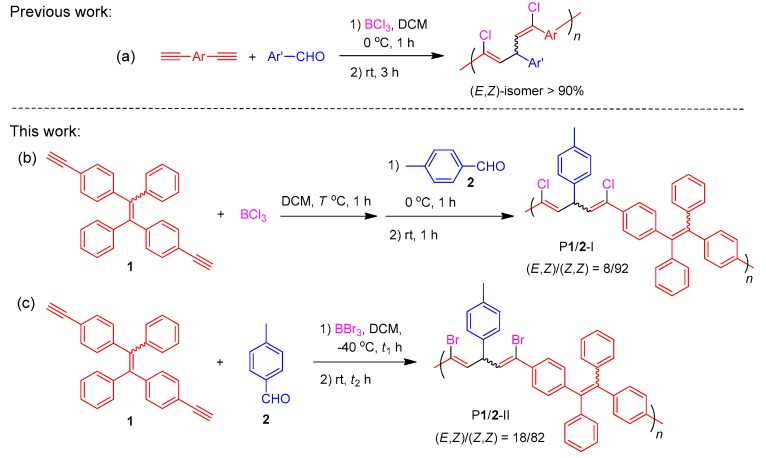
Metal-free BX_3_-mediated multicomponent polymerizations (MCP) of alkyne and aldehyde toward stereo-defined poly(1,4-pentadiene)s: (**a**) BCl_3_-mediated MCP toward poly(1,4-pentadiene)s with dominant (*E*,*Z*)-isomeric structures; (**b**) BCl_3_- and (**c**) BBr_3_-mediated MCP toward poly(1,4-pentadiene)s with dominant (*Z*,*Z*)-isomeric structures.

Recently, an interesting boron trihalide (BX_3_)-mediated alkyne-aldehyde coupling reaction toward stereo-defined 1,3,5-triaryl-1,5-dihalo-1,4-pentadienes (Scheme 1) have attracted our great interest [39,40]. The stereoselectivity of this reaction can be well tuned by simply changing the reaction conditions or the type of BX_3_ employed. In the presence of boron trichloride (BCl_3_), the reactions of aryl aldehydes with 2 equivalents of arylacetylenes afforded (*E*,*Z*)-1,4-pentadiene as a major product with a minor quantity of the (*Z*,*Z*)-isomer (Scheme 1, route a) [39]. By controlling the reaction temperature and the sequence of reactant addition, (*Z*,*Z*)-1,5-dichloro-1,4-pentadiene was obtained as the major product (Scheme 1, route b) [40]. When boron tribromide (BBr_3_) was used as the mediator at low temperature, the corresponding (*Z*,*Z*)-1,3,5-triaryl-1,5-dibromo-1,4-pentadienes were formed as the primary products in excellent yields (Scheme 1, route c) [39]. Previously, we have developed Scheme 1a into a metal-free MCP for the stereoselective construction of polymers with mainly (*E*,*Z*)-1,5-dichloro-1,4-pentadiene units (Scheme 2, route a) [41]. Due to our continuing interest in the development of new polymerization reactions to synthesize stereoregular functional polymers, herein we intend to further explore the possibility of developing the MCRs in Scheme 1b,c into efficient stereoselective MCPs for the construction of poly(pentadiene)s with dominant (*Z*,*Z*)-isomeric structures.

By varying the polymerization conditions and the types of boron trihalide used, in this work, we successfully prepared two stereo-defined functional polymers with (*Z*,*Z*)-1,4-pentadiene as major segments (Scheme 2b,c) by the boron trihalide-mediated polycoupling reactions of tetraphenylethylene (TPE)-containing terminal diyne (**1**) and *p*-tolaldehyde (**2**). Polymer P**1**/**2**-I with (*Z*,*Z*)-1,5-dichloropentadiene as the major isomeric structure was obtained by changing the addition sequence of the reactants to allow the diyne monomer to react with BCl_3_ first in refluxed dichloromethane (DCM) (Scheme 2, route b). On the other hand, polymer P**1**/**2**-II dominated with (*Z*,*Z*)-1,5-dibromopentadiene unit was generated by replacing BCl_3_ with BBr_3_ at low polymerization temperature of −40 °C (Scheme 2, route c). Both polymers possess excellent film-forming ability and their thin films show high refractive index and low chromatic dispersion. The TPE units of the polymers endow them with much stronger fluorescence in the aggregate state than in the solution state, showing an obvious aggregation-enhanced emission (AEE) property. Considering the significance of explosive detection in homeland security and antiterrorism, we also investigated the possibility of utilizing the fluorescent nanoaggregates of the obtained AEE polymers as fluorescent chemosensors for the sensitive detection of explosives.

## 2. Experimental Section

### 2.1. Materials and Methods

Monomer **1** was synthesized according to the reported procedures [42]. Monomer **2**, BCl_3_, BBr_3_ and all other reagents used for monomer synthesis and polymerizations were purchased from Aldrich (Hong Kong, China) and used without further purification. Tetrahydrofuran (THF) was distilled from sodium benzophenone ketyl under nitrogen immediately prior to use. Dichloromethane (DCM) was purchased from Aldrich and distilled from molecular sieves under nitrogen prior to use. Weight-average molecular weights (*M*_w_) and polydispersity indices (PDI) of the obtained polymers were estimated by a Waters gel permeation chromatography (GPC) system equipped with a Waters 515 HPLC pump (Waters, Hong Kong, China), a set of Styragel columns (HT3, HT4 and HT6; *M*_W_ range 10^2^–10^7^), a column temperature controller, a Waters 486 wavelength-tunable UV-vis detector and a Waters 2414 differential refractometer (Waters, Hong Kong, China). THF was used as eluent at a flow rate of 1.0 mL/min. The column temperature was maintained at 40 °C and the working wavelength of the UV-vis detector was set at 254 nm. A set of monodisperse polystyrene standards (Waters) covering a *M*_W_ range of 10^3^–10^7^ were used for *M*_w_ calibration. The THF solutions of the polymer samples (~1 mg/mL) used for GPC measurement were filtered through 0.45 μm PTFE syringe-type filters before being injected into the GPC system. FT-IR spectra were recorded on a Bruker Vertex 70 FT-IR spectrometer using KBr disk (Bruker, Hong Kong, China). ^1^H and ^13^C NMR spectra were measured on a Bruker ARX 400 NMR spectrometer in deuterated chloroform (chloroform-*d*). Chemical shifts were calibrated using CDCl_3_ as internal reference at *δ* 7.26 (^1^H NMR) and *δ* 77.16 (^13^C NMR). Thermogravimetric analysis (TGA) was carried on a Perkin-Elmer TGA 7 analyzer (PerkinElmer, Hong Kong, China) at a heating rate of 10 °C/min under nitrogen. UV-vis spectra and photoluminescence (PL) spectra were recorded on a PerkinElmer Lambda 365 UV/Vis spectrophotometer and a Horiba Fluorolog spectrophotometer (Horiba, Hong Kong, China), respectively. Fluorescence quantum yields were measured using a Hamamatsu absolute PL quantum yield spectrometer C11347 Quantaurus_QY (Hamamatsu Photonics, Hong Kong, China). Refractive indices (RI or *n*) were determined on a J.A. Woollam Variable Angle Ellipsometer (J.A. Woollam, Hong Kong, China), with a model of Alpha-SE and a wavelength tunability from 380 to 890 nm. The polymer thin films for RI measurement were prepared by spin-coating the 1,2-dichloroethane solutions of polymer samples (~10 mg/mL) at 1000 rpm for 1 min on silicon wafers and then dried in a vacuum oven at room temperature overnight.

### 2.2. Polymerization

Preparation procedures of P**1**/**2**-I: Into a 15 mL Schlenk tube with a stirring bar was added diyne **1** (0.20 mmol, 76 mg). The tube was evacuated under vacuum and then flushed with nitrogen for three cycles. Afterward, 2 mL of distilled DCM was injected into the tube followed by the dropwise addition of a solution of BCl_3_ in hexane (1.0 M, 0.22 mL, 0.22 mmol) under nitrogen. The reaction mixture was heated to 45 °C and refluxed for 1 h under stirring. Then the reaction tube was cooled to 0 °C using an ice bath and *p*-tolualdehyde **2** (0.20 mmol, 23.6 μL) was added into the reaction system under the protection of nitrogen gas. The solution mixture was first stirred at 0 °C for 1 h and then warmed to room temperature to stir for another 1 h. The reaction mixture was then quenched by a small amount of water with continuous stirring and diluted with THF. Subsequently, the resulting solution was added dropwise to a 200 mL of methanol via a cotton filter under vigorous stirring. The precipitates were allowed to stand overnight before being collected by filtration and washed with methanol and hexane. The obtained polymer was dried under vacuum at room temperature to a constant weight. Characterization data: yellow powder; yield 68%. *M*_w_: 119,700; *M*_w_/*M*_n_: 3.9 (GPC, polystyrene calibration). IR (KBr), *ν* (cm^−1^): 3051, 3023, 2921, 2860, 2818, 1946, 1898, 1802, 1686, 1599, 1503, 1443, 1402, 1308, 1243, 1186, 1075, 1019. ^1^H NMR (CDCl_3_, 400 MHz), *δ* (ppm): 7.35–6.98, 6.27, 5.36, 2.32. ^13^C NMR (CDCl_3_, 100 MHz), *δ* (ppm): 144.29, 143.49, 143.35, 140.78, 138.47, 137.76, 137.52, 136.59, 135.68, 133.91, 131.44, 131.39, 129.61, 129.41, 128.06, 127.86, 127.42, 127.24, 126.94, 126.68, 126.11, 125.91, 45.60, 21.18.

P**1**/**2**-II: To a 15 mL Schlenk tube with a stirring bar was added diyne **1** (0.20 mmol, 76 mg). A vacuum-nitrogen cycle was then performed three times before 2 mL of distilled DCM was injected by syringe followed by the addition of *p*-tolualdehyde **2** (0.20 mmol, 23.6 μL). Subsequently, the mixture was cooled to −40 °C in a dry ice/acetonitrile cooling bath and the solution of BBr_3_ in DCM (1.0 M, 0.22 mL, 0.22 mmol) was then dropwise added into the mixture. The solution was stirred at −40 °C for 2 h and then warmed to room temperature and stirred for another 1 h. The reaction mixture was then terminated by adding a small amount of water and diluted with THF. The resulting solution was then added dropwise to a 200 mL of methanol via a cotton filter with vigorous stirring to precipitate the polymeric products. The precipitates were allowed to stand overnight and then collected after filtration, washing with methanol and hexane and dried under vacuum at room temperature to a constant weight. Characterization data: brownish yellow powder; yield 79%. *M*_w_: 63,100; *M*_w_/*M*_n_: 4.3 (GPC, polystyrene calibration). IR (KBr), *ν* (cm^−1^): 3051, 3022, 2920, 2860, 2818, 1946, 1899, 1685, 1599, 1510, 1492, 1443, 1403, 1356, 1267, 1182, 1111, 1074, 1019. ^1^H NMR (CDCl_3_, 400 MHz), *δ* (ppm): 7.31–7.00, 6.38, 5.25, 2.32. ^13^C NMR (CDCl_3_, 100 MHz), *δ* (ppm): 144.30, 143.54, 143.33, 140.75, 137.98, 137.44, 137.33, 136.69, 131.45, 131.33, 129.63, 128.57, 128.06, 127.85, 127.46, 127.25, 127.07, 126.95, 126.87, 51.31, 21.19.

To assist the structural characterization of P**1**/**2**-I and P**1**/**2**-II, polymer P**1**/**2** was synthesized by the polymerization route shown in Scheme 2a and Scheme S1 using TPE-containing diyne **1**, *p*-tolualdehyde **2** and BCl_3_ as reactants. According the reported procedures in the literature [41], **1** (0.2 mmol, 76.0 mg) was first added into a 15 mL Schlenk tube equipped with a stirring bar and then the tube was evacuated under vacuum and flushed with dry nitrogen for three times. Subsequently, 2 mL of distilled DCM and aldehyde **2** (0.20 mmol, 23.6 μL) were injected into the reaction system under nitrogen atmosphere. The mixture was then cooled to 0 °C followed by the dropwise addition of 0.22 mL of BCl_3_ solution (1.0 M in hexane, 0.22 mmol). The solution mixture was stirred at 0 °C under nitrogen for 1 h and then was warmed to room temperature to stir for another 3 h. After the reaction mixture was quenched with water and diluted with THF, the resulting solution was added dropwise to a 200 mL of methanol via a cotton filter under vigorous stirring. The precipitates were collected by filtration, washing with methanol and hexane, and then drying under vacuum at room temperature to a constant weight. IR (KBr), *ν* (cm^−1^): 3076, 3052, 3023, 2922, 2861, 1946, 1898, 1798, 1685, 1623, 1599, 1503, 1441, 1401, 1349, 1308, 1242, 1184, 1113, 1074, 1019. ^1^H NMR (CDCl_3_, 400 MHz), *δ* (ppm): 7.35–7.01, 6.20–6.07, 4.73, 2.33. ^13^C NMR (CDCl_3_, 100 MHz), *δ* (ppm): 144.44, 144.34, 143.54, 143.34, 143.17, 141.02, 140.74, 138.73, 136.69, 135.49, 135.00, 134.89, 133.36, 132.63, 131.45, 131.28, 129.64, 129.13, 128.46, 128.25, 128.07, 128.01, 127.86, 127.24, 126.96, 126.82, 125.86, 45.47, 21.17.

### 2.3. Preparation of Model Compounds

The model compounds (**3**–**5**) were prepared according to the literature methods [39,40]. Their characterization data are given as follows.

1,5-Dichloro-1,5-diphenyl-3-(*p*-tolyl)-(*E*,*Z*)-1,4-pentadiene (**3**) [39]: ^1^H NMR (CDCl_3_, 400 MHz): *δ* 7.58–7.15 (m, 14H), 6.26 (d, 1H, *J* = 9.2 Hz), 6.18 (d, 1H, *J* = 10.6 Hz), 4.84 (t, 1H, *J* = 10.0 Hz), 2.33 (s, 3H). ^13^C NMR (CDCl_3_, 100 MHz): *δ* 138.5, 137.6, 136.7, 136.6, 133.5, 132.8, 129.5, 129.2, 128.9, 128.8, 128.3, 127.8, 127.1, 126.5, 45.2, 21.0.

1,5-Dichloro-1,3,5-triphenyl-(*Z*,*Z*)-1,4-pentadiene (**4**) [40]: ^1^H NMR (chloroform-*d*): *δ* 7.63–7.24 (m, 15H), 6.34 (d, 2H, *J* = 9.0 Hz), 5.47 (t, 1H, *J* = 9.0 Hz). ^13^C NMR (chloroform-*d*): *δ* 141.3, 137.7, 134.3, 128.8, 128.7, 128.3, 127.4, 127.3, 126.9, 126.6, 45.7.

1,5-Dibromo-1,5-diphenyl-3-(*p*-tolyl)-(*Z*,*Z*)-1,4-pentadiene (**5**) [39]: ^1^H NMR (CDCl_3_, 400 MHz): *δ* 7.58–7.14 (m, 14H), 6.43 (d, 2H, *J* = 8.9 Hz), 5.30 (t, 1H, *J* = 8.9 Hz), 2.33 (s, 3H). ^13^C NMR (CDCl_3_, 100 MHz): *δ* 139.6, 137.8, 136.5, 131.2, 129.5, 128.7, 128.2, 127.7, 127.3, 126.8, 51.0, 21.0. 

## 3. Results and Discussion

### 3.1. Polymer Synthesis

In order to develop the MCRs shown in Scheme 1b,c into polymerization tools for the construction of functional polymers with dominant (*Z*,*Z*)-1,4-pentadiene isomeric structures, terminal diyne **1** with TPE moiety was first synthesized according to the literature [42]. To obtain soluble polymers with high molecular weights, the derived BX_3_-mediated polymerizations of **1** and *p*-tolualdehyde **2** were carried out under different conditions and the polymerization results were summarized in Table 1 and Table 2.

In our previous work, we have systematically studied the key parameters involved in the multicomponent polymerization of **1**, **2** and BCl_3_ for the synthesis of P**1**/**2** with (*E*,*Z*)-diene as major structural segments [41]. The best polymerization result was obtained by first reacting in DCM at 0 °C for 1 h followed by a further reaction at room temperature for 3 h at a monomer concentration of [1]:[2]:[BCl_3_] = 0.1:0.1:0.11. Based on the experience in the synthesis of P**1**/**2** (Scheme S1) and the small molecular reactions shown in Scheme 1b, herein we tried to react **1** with BCl_3_ in DCM at a reflux temperature prior to coupling with **2** at 0 °C. At first, the reflux temperature was set as *T* = 55 °C and the reaction mixture of **1** and BCl_3_ was stirred at this temperature for 1 h followed by the addition of **2** at 0 °C. After stirring at 0 °C for 1 h and at room temperature for another 1 h, an insoluble gel was, however, formed (Table 1, no. 1). We then decreased the reflux temperature to *T* = 45 °C while keeping other polymerization conditions unchanged (Table 1, no. 2). Under such conditions, soluble P**1**/**2**-I with a high molecular weight of 119,700 was obtained in a satisfactory yield (68%). Regarding the BBr_3_-mediated alkyne-aldehyde coupling reaction (Scheme 1c), the derived polymerization of **1** and **2** was first carried out at −40 °C due to the reported high reaction efficiency of the bromination process of aryl aldehydes by tribromoborane [39,43]. Upon the addition of BBr_3_ to the DCM solution of **1** and **2** at −40 °C, the reaction was kept stirring at the same temperature for 4 h (*t*_1_ = 4 h). However, only oligomers with a molecular weight of 6900 were collected (Table 2, no. 1). We then allowed the polymerization to react for an extra hour at room temperature (*t*_2_ = 1 h), but gelation process occurred under this condition (Table 2, no. 2). These results suggest that the reaction could be readily triggered at −40 °C [39,40] and the polymer chains grow faster at room temperature. When the the reaction time at −40 °C was shortened to 2 h (*t*_1_ = 2 h), a soluble polymer P**1**/**2**-II with a high molecular weight of 63,100 was afforded in a good yield of 79% (Table 2, no. 3). Further decreasing *t*_1_ to 1 h lead to a slightly lower molecular weight and isolated yield (Table 2, no. 4).

### 3.2. Structural Characterization

**Scheme 3:**
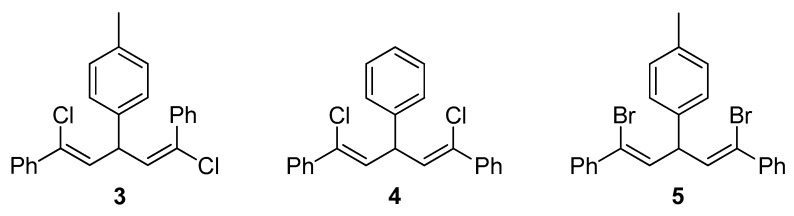
Chemical structures of model compounds **3**, **4** and **5**.

To assist the structural characterization of the obtained polymers, stereoregular poly[1,5-dichloro-(*E*,*Z*)-1,4-pentadiene] P**1**/**2** was prepared according to the literature method (Scheme S1) [41]. The characterization data of compounds **3**, **4** and **5** (Scheme 3) reported by Kabalka and co-workers [39,40] were utilized to assist further analysis on the stereochemistry of P**1**/**2**, P**1**/**2**-I and P**1**/**2**-II, respectively.

**Figure 1:**
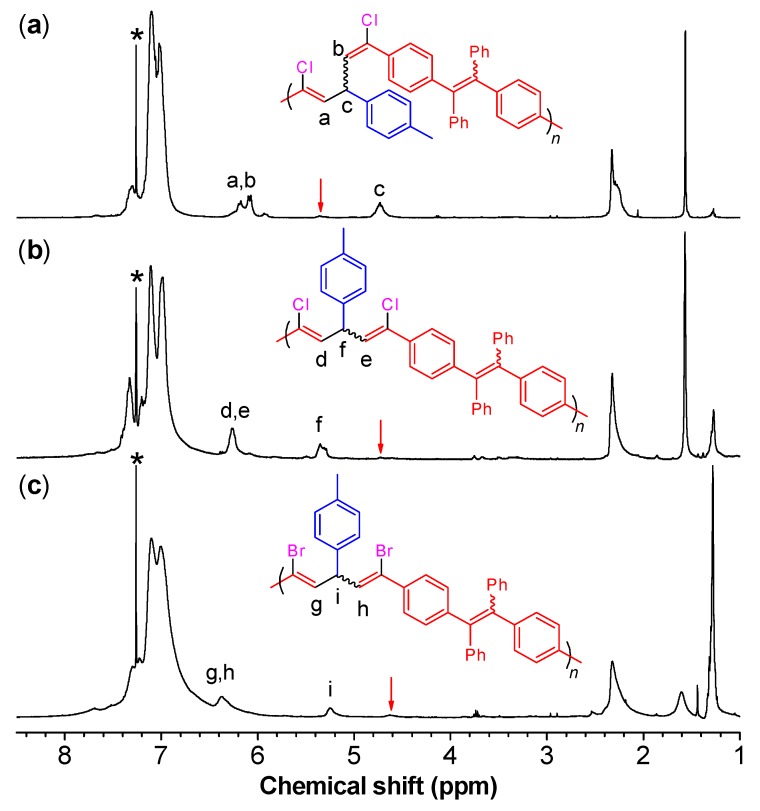
^1^H NMR spectra of (**a**) P**1**/**2**; (**b**) P**1**/**2**-I (Table 1, no. 2) and (**c**) P**1**/**2**-II (Table 2, no. 3) in chloroform-*d*. The peaks related to chloroform-*d* are marked with asterisks.

As indicated by the ^1^H NMR results of model compound **3** (see the Experimental section) and P**1**/**2** (Figure 1a), the vinyl protons of the 1,5-dichloro-(*E*,*Z*)-1,4-pentadiene show two resonance peaks due to their slight difference in the chemical environment. The vinyl proton “a” of *Z*-configuration resonates at a relatively lower field of *δ* 6.26 than that of *E*-configuration (“b”; *δ* 6.18). Meanwhile, the resonance peak of the methyne proton “c” of P**1**/**2** was observed at *δ* 4.84 in Figure 1a accompanied with an almost invisible peak at *δ* ~5.30. By comparing with the characterization results of **3** and **4**, the peaks at *δ* 4.84 and *δ* 5.30 are attributed to the resonances of the (*E*,*Z*)- and (*Z*,*Z*)-1,4-pentadiene units, respectively. By integrating the “c” peak and the tiny peak at *δ* 5.30, the ratio of (*E*,*Z*)- to (*Z*,*Z*)-conformers in P**1**/**2** was calculated to be 95/5.

On the other hand, the vinyl protons of 1,5-dihalo-(*Z*,*Z*)-1,4-pentadiene (**4** and **5**) show only one resonance peak due to their symmetric structures. The vinyl protons of **4** resonate at *δ* 6.34, while the peak of its methyne proton emerges at *δ* 5.47. In the ^1^H NMR spectrum of P**1**/**2**-I as shown in Figure 1b, a sole peak corresponding to the absorption of the two vinyl protons of 1,5-dicloro-(*Z*,*Z*)-1,4-pentadiene skeleton (“d” and “e”) of P**1**/**2**-I was observed at *δ* 6.27, which is consistent with the characterization result of **4**. The signal of the methyne proton “f” of P**1**/**2**-I, on the other hand, was observed at *δ* 5.35 in Figure 1b accompanied with an almost invisible peak at *δ* 4.73. By integrating the “f” peak and this tiny peak, the ratio of (*Z*,*Z*)- to (*E*,*Z*)-conformers was calculated to be 92/8. Similarly, the vinyl protons and methyne proton of 1,5-dibromo-(*Z*,*Z*)-1,4-pentadiene (**5**) resonates at *δ* 6.43 and *δ* 5.30, respectively. Correspondingly, the ^1^H NMR spectrum of P**1**/**2**-II shows a single broad peak related to the absorption of the two vinyl protons of the 1,5-dibromo-(*Z*,*Z*)-1,4-pentadiene skeleton (“g” and “h”) at *δ* 6.37 (Figure 1c). The resonance peak of the methyne proton “i” locates at *δ* 5.25 with a minor peak at *δ* 4.63. The ratio of (*Z*,*Z*)- to (*E*,*Z*)-conformers of P**1**/**2**-II was calculated to be 82/18. These results clearly suggest that P**1**/**2**-I and P**1**/**2**-II are predominantly composed of (*Z*,*Z*)-1,4-pentadiene units.

**Figure 2:**
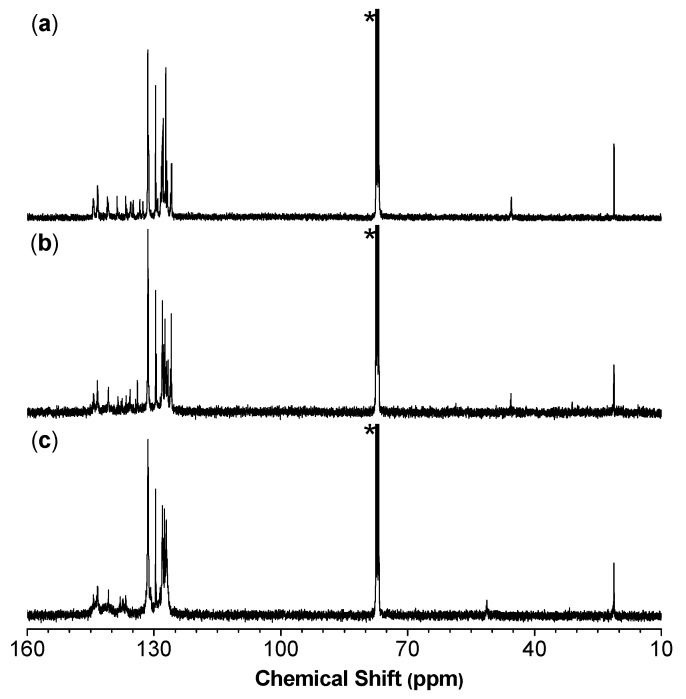
^13^C NMR spectra of (**a**) P**1**/**2**; (**b**) P**1**/**2**-I (Table 1, no. 2) and (**c**) P**1**/**2**-II (Table 2, no. 3) in chloroform-*d.* The peaks related to chloroform-*d* are marked with asterisks.

Analysis by ^13^C NMR further support the conclusion drawn from the ^1^H NMR results. As shown in Figure 2, an obvious resonance peak at *δ* 45.4, 45.5, and 51.2 was observed in the ^13^C NMR spectrum of P**1**/**2**, P**1**/**2**-I, and P**1**/**2**-II, respectively. This result is consistent with the characteristic resonance signal of the methyne carbon of 1,5-dichloro-(*E*,*Z*)-1,4-pentadiene (**3**), 1,5-dichloro-(*Z*,*Z*)-1,4-pentadiene (**4**), and 1,5-dibromo-(*Z*,*Z*)-1,4-pentadiene (**5**), which appears at *δ* 45.2, 45.7, and 51.0, respectively [39,40]. The structure of P**1**/**2** has been fully confirmed in our previous publication by comparing its characterization results with those of the monomers and model compound **3** [41]. As the IR and NMR spectra of P**1**/**2**-I and P**1**/**2**-II largely resemble those of P**1**/**2** apart from the above-mentioned difference induced by isomeric structures, they should contain the same 1,3,5-triaryl-1,5-dihalo-1,4-pentadiene units but with (*Z*,*Z*)-configuration as the predominant structure.

### 3.3. Solubility and Thermal Stability

**Figure 3:**
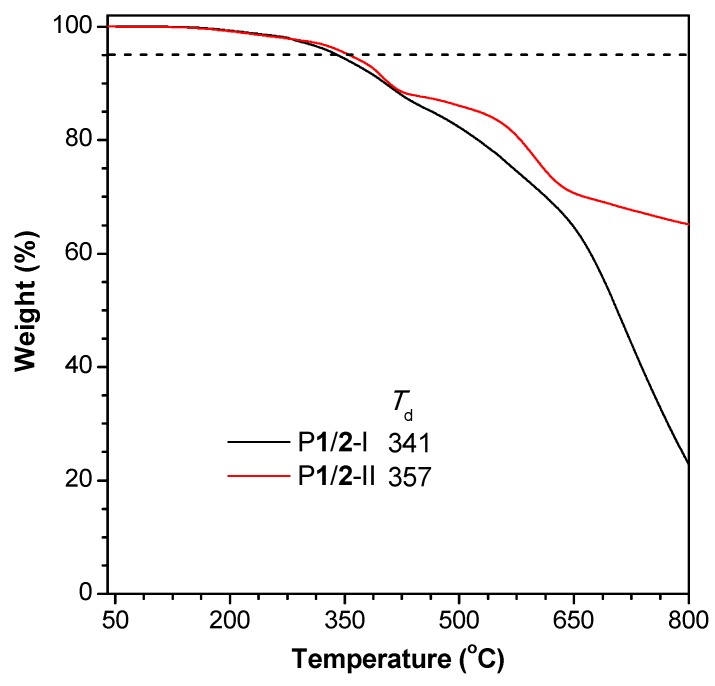
TGA thermograms of P**1**/**2**-I and P**1**/**2**-II recorded under nitrogen at a heating rate of 10 °C/min. *T*_d_ represents the degradation temperature for 5% weight loss.

The twisted conformation of the TPE moieties and the flexible 1,4-pentadiene structures endow P**1**/**2**-I and P**1**/**2**-II with good solubility in common organic solvents, such as THF, DCM, chloroform, and 1,2-dichloroethane. The thermal stability of the obtained polymers was evaluated by TGA under nitrogen. The results shown in Figure 3 suggest that P**1**/**2**-I and P**1**/**2**-II exhibit excellent thermal stability, losing merely 5% of their weights at a high temperature (*T*_d_) of 341 °C and 357 °C, respectively. It is worth noting that P**1**/**2**-II retains more than 60% of its original weight after being heated to 800 °C under nitrogen. The high *T*_d_ and char yield of P**1**/**2**-I and P**1**/**2**-II enable them to find potential high-tech applications as heat-resistant materials [44,45,46].

### 3.4. Light Refraction and Chromatic Dispersion

**Figure 4:**
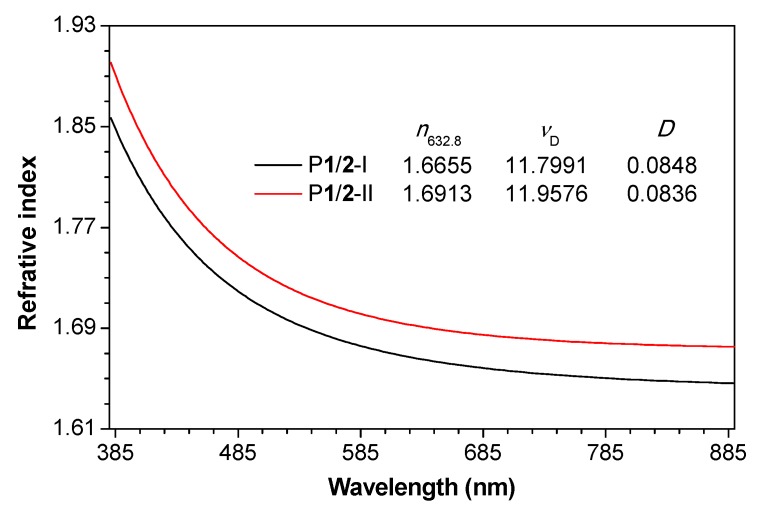
Wavelength dependence of refractive indices of thin films of P**1**/**2**-I and P**1**/**2**-II. Abbreviation: *n* = refractive index, *ν*_D_ = Abbé number = (*n*_D_ − 1)/(*n*_F_ − *n*_C_), where *n*_D_, *n*_F_ and *n*_C_ are the RI values at wavelengths of 589.2, 486.1 and 656.3 nm, respectively, *D* = chromatic dispersion = 1/*ν*_D_.

Processable polymeric materials with high refractive index (*n*) are in great demand due to their potential applications in the fabrication of advanced photonic and optoelectronic devices [47,48]. For instance, the *n* value of polymer microlens used for image sensors of complementary metal oxide semiconductor often exceeds 1.70 or even 1.80 [47]. However, typical *n* values of conventional optical plastics such as polystyrene, poly(methyl methacrylate) and polycarbonate generally fall in the range of 1.30–1.59 [49], which prevent them to be applied as advanced optical materials. Theoretically, polymers with aromatic rings and halogen atoms tend to show high *n* values [47,48,50]. Therefore, P**1**/**2**-I and P**1**/**2**-II are likely to be high-*n* materials. The good solution-processability and film-forming ability of these two polymers enable us to investigate the light refraction of their thin films. Tough polymer thin films can be readily fabricated by spin-coating their 1,2-dichloroethane solutions on silica wafers. The results shown in Figure 4 reveal that both P**1**/**2**-I and P**1**/**2**-II exhibit high *n* values of 1.9007–1.6753 and 1.8570–1.6462, respectively, in a wide wavelength region of 380–890 nm. The *n* value of P**1**/**2**-I and P**1**/**2**-II at 632.8 nm is 1.6655 and 1.6913, respectively, which is much higher than those of conventional optical plastics. The relatively higher *n* values of P**1**/**2**-II than P**1**/**2**-I can be explained by the intrinsically higher molar refraction of bromine than chloride atom [47].

In addition to refractive index, chromatic dispersion (*D*) is another critical parameter for optical materials [51]. *D* is defined as the reciprocal of Abbé number (*v*_D_) and reflects the wavelength dependence of the *n* values of a material [52]. For an optical material to be used in practical applications such as serving as lenses for ophthalmological application, both high *n* values and low *D* (or high *v*_D_) are required [53]. As illustrated in Figure 4, both P**1**/**2**-I and P**1**/**2**-II show a similarly low optical dispersion. Their *v*_D_ value is 11.7991 and 11.9576, respectively, and the corresponding *D* value is 0.0848 and 0.0836, respectively.

### 3.5. Aggregation-Enhanced Emission

Luminescent polymeric materials with smart features have attracted tremendous interest due to their wide applications in bio-imaging, optical data storage, fluorescent chemo-/biosensors, light-emitting devices, etc. [54,55,56,57,58,59]. Conventional luminescent polymers are often composed of chromophores with large planar conformation. Such a structural feature often leads to the problem of emission quenching in the solid or aggregate state to greatly limit their practical applications [60]. In 2001, our group proposed a concept of aggregation-induced emission (AIE), which is the exact opposite of the aforementioned aggregation-caused quenching effect [61]. Luminogens with AIE attributes (AIEgens) are non-emissive in dilute solutions but become highly emissive upon aggregate formation [62,63]. The unique AIE effect enables AIE-active polymers to find various high-tech applications as thin films or in aqueous media [64,65,66].

**Figure 5:**
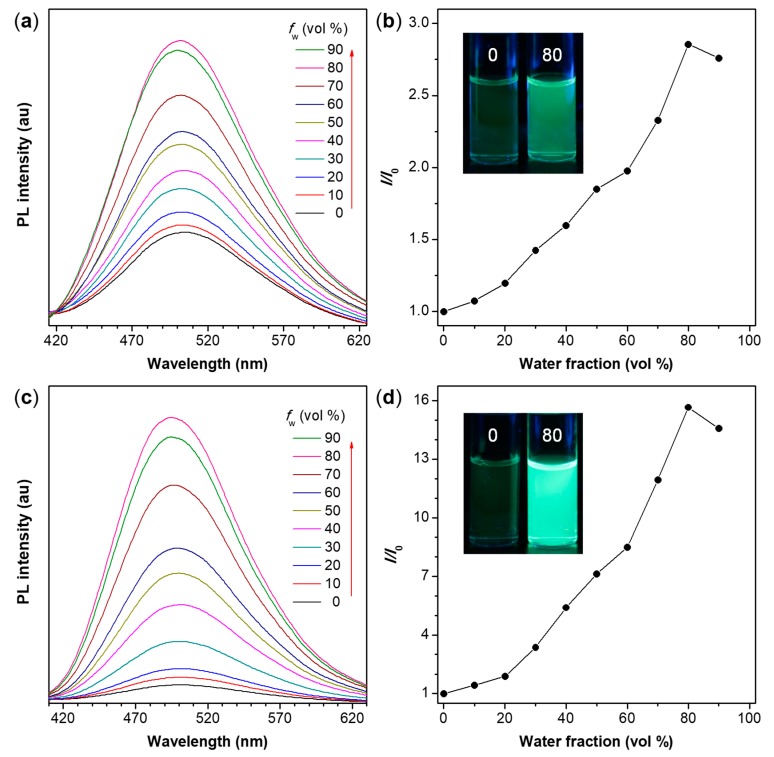
(**a**,**c**) PL spectra of (**a**) P**1**/**2**-I and (**c**) P**1**/**2**-II in THF and THF/water mixtures with different water fractions (*f*_w_); (**b**,**d**) Plot of relative emission intensity (*I*/*I*_0_) versus the composition of the aqueous mixture of (**b**) P**1**/**2**-I and (**d**) P**1**/**2**-II, where *I* = peak intensity and *I*_0_ = peak intensity in pure THF solution. Inset: fluorescent photographs of P**1**/**2**-I and P**1**/**2**-II in pure THF solution and in THF/water mixture with *f*_w_ of 80% taken under 365 nm UV illumination from a hand-held UV lamp. Solution concentration: 10 μM; excitation wavelength: 350 nm.

TPE is a well-known AIEgen [67]. Therefore, the obtained TPE-containing polymers P**1**/**2**-I and P**1**/**2**-II are also anticipated to be AIE-active. The absorption spectra of these two polymers in THF were first measured before studying their photoluminescence (PL) properties. As depicted in Figure S1, P**1**/**2**-I and P**1**/**2**-II exhibit a similar absorption spectral pattern with an absorption maximum at 340 nm due to their similar molecular structures. To verify whether they are the AIE-active, we systematically studied their PL behaviors in THF/water mixtures with different water fractions (*f*_w_). The results shown in Figure 5 and Figure S2 suggest that the present polymers P**1**/**2**-I and P**1**/**2**-II show a phenomenon of aggregation-enhanced emission (AEE). As shown in Figure 5a,b, the dilute THF solution of P**1**/**2**-I is weakly emissive. However, its PL intensity is gradually enhanced when an increasing amount of water, a poor solvent for the polymer samples, is added to the THF solution to induce the formation of aggregates. The PL intensity reaches its maximum at *f*_w_ of 80%. The slight drop in the PL intensity at *f*_w_ of 90% is possibly owing to the formation of extensive aggregates to make the suspension unstable and lower the effective solute concentration [68]. P**1**/**2**-II shows similar PL behaviors. The PL intensity of P**1**/**2**-II starts to rise upon water addition and keeps increasing as the *f*_w_ increases to 80% (Figure 5c). The highest PL intensity is attained at *f*_w_ of 80%, being about 160-fold higher than that in pure THF solution (Figure 5d). The dependence of the fluorescence quantum yield (*Ф*_F_) of P**1**/**2**-I and P**1**/**2**-II on the solvent composition of the THF/water mixture (Figure S2) also confirmed their AEE characteristics. The dilute THF solutions of P**1**/**2**-I and P**1**/**2**-II show a low *Ф*_F_ of 0.8%. With the gradual addition of water, their *Ф*_F_ value continuously increase and reach a maximum of 3.1% and 9.4%, respectively. Nanoaggregates with sizes in the range of 345–271 nm and 323–217 nm are detected in aqueous solutions of P**1**/**2**-I and P**1**/**2**-II, respectively (Figures S3 and S4) by dynamic light scattering measurements. The aggregates of P**1**/**2**-I and P**1**/**2**-II show blue-green emission with emission maximum at around 500 nm.

The restriction of intramolecular motion (RIM) has been proposed to be the main cause of the AIE phenomenon of TPE [62,69]. Similar to TPE, the RIM mechanism is proposed to play an important role for the AEE properties of P**1**/**2**-I and P**1**/**2**-II. In the solution state, the phenyl rings in the polymer structures can freely rotate or twist against the ethene stators. Such active intramolecular motions serve as nonradiative channels for the excited state to decay to the ground state to quench the emission. Different from the isolated TPE molecules, the TPE units are covalently linked in the polymer structures and thus the motions of the phenyl rings are restricted to some extent. Therefore, the dilute solutions of P**1**/**2**-I and P**1**/**2**-II are emissive although the emission is very weak. When the polymers form aggregates in the presence of a large amount of water, the intramolecular motions are further restricted due to the involved physical constraint, which blocks the nonradiative relaxation channels and hence enhances the light emission of the polymers.

### 3.6. Explosive Detection

Sensitive detection of nitroaromatic-based explosives, such as picric acid (PA), 2,4-dinitrotoluene and 2,4,6-trinitrotoluene, is of great significance in homeland security and environmental assays and has antiterrorism implications. Fluorescent chemosensors based on AIE-active polymers generally have the advantages of high stability, fast and amplified response, and superior sensitivity to analytes over their small molecular counterparts [65]. They can serve as solid-state detectors or be used in aqueous media without suffering from the problem of analyte-induced self-quenching. To demonstrate the potential of the obtained AIE polymers for explosive detection, picric acid (PA) was selected as a model analyte in this work for its commercial availability. Dispersed nanoaggregates of P**1**/**2**-I and P**1**/**2**-II were prepared in their THF/water mixtures with 80% water fraction and used as fluorescent probes. The change in the PL properties of the polymer aggregates in response to PA was quantitively monitored by measuring the PL spectra of the polymer aggregates in the presence of different amounts of PA.

**Figure 6:**
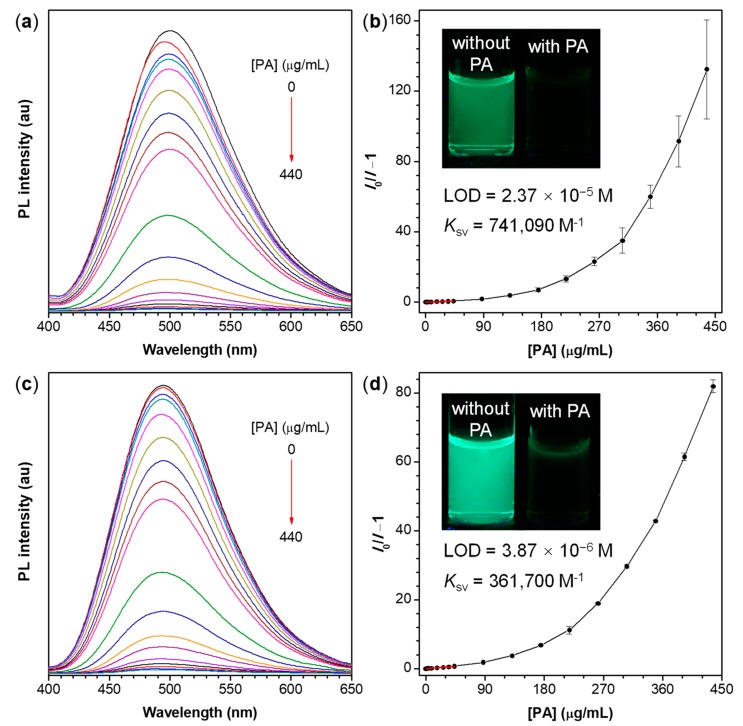
(**a**,**c**) PL spectra of (**a**) P**1**/**2**-I and (**c**) P**1**/**2**-II in THF/water mixtures with 80% water fraction containing different amounts of picric acid (PA); (**b**,**d**) Stern-Volmer plots of (*I*_0_/*I*−1) of (**b**) P**1**/**2**-I and (**d**) P**1**/**2**-II versus the PA concentration, where *I* = peak intensity and *I*_0_ = peak intensity without PA. Solution concentration: 10 μM; excitation wavelength: 350 nm. Inset in panel (**b**,**d**): fluorescent photographs of P**1**/**2**-I and P**1**/**2**-II in THF/water mixture with *f*_w_ of 80% without and with PA taken under 365 nm UV illumination from a hand-held UV lamp.

As shown in Figure 6, with the gradual addition of PA into the aqueous mixture of the polymers, the PL intensity of the polymer aggregates decreases progressively while the PL spectral profile remains almost unchanged. At a PA concentration of 0.44 mg/mL, the fluorescence of P**1**/**2**-I and P**1**/**2**-II is almost completely quenched and their PL intensity (*I*) decreases by more than 99% compared with that in the absence of PA (*I*_0_). Different from the conventional linear Stern-Volmer plots of sensors working in solutions [70], the Stern−Volmer plots of the relative PL intensity (*I*_0_/*I*−1) of P**1**/**2**-I and P**1**/**2**-II aggregates versus the PA concentration are close to linear at first when the PA concentration is lower than 44 μM but subsequently bend upward with the further increase of PA content (Figure 6b,d). This result indicates that the quenching process of these two polymer nanoaggregate systems becomes more efficient at higher quencher concentration and shows a superamplification quenching effect [71]. As shown in Figures S5 and S6, large quenching constants (*K*_sv_) of up to 741,090 and 361,700 M^−1^ are deducted from the Stern−Volmer plots of P**1**/**2**-I and P**1**/**2**-II, respectively. These values are much higher than those of chemosensors based on polyindole/CdS nanocomposites [72], polysiloles and polygermoles in solutions [73], and small molecules such as benzimidazoliums [74], phthalocyanines [75], and quinoxaline derivatives [76,77], whose *K*_sv_ falls in the range of 6710–32,900 M^−1^. According to the equation of LOD = 3 SB/m, the limit of detection (LOD) values of P**1**/**2**-I and P**1**/**2**-II aggregates toward PA are calculated to be 2.37 × 10^−5^ and 3.87 × 10^−6^ M, respectively, and are comparable to those of the previous reports [78,79,80]. The parameter of SB in the aforementioned equation refers to the standard deviation of the blank measurement and m represents the slope of the linear region of the Stern-Volmer plot [81]. These results demonstrate that the nanoaggregate suspensions of P**1**/**2**-I and P**1**/**2**-II can be utilized as promising fluorescent chemo-sensors with high sensitivity, amplified response and low detection limit for explosive detection.

To investigate the quenching mechanism, the absorption spectra of PA was measured and compared with the PL spectra of P**1**/**2**-I and P**1**/**2**-II nanoaggregates in 80% aqueous mixture. As shown in Figure S7, the absorption spectrum of PA is partially overlapped with the PL spectra of the polymer nanoaggregates. This result suggests that effective energy transfer can readily occur from the excited state of the polymer aggregates to the ground state of PA at an appropriate distance to quench the fluorescence of the polymers through nonradiative relaxation process [82,83]. Moreover, PA is a typical electron-deficient compound due to its highly nitrated structure, whereas both P**1**/**2**-I and P**1**/**2**-II possess an electron-rich backbone. The potential involvement of Lewis acid-base interactions between the electron-deficient analytes and the electron-rich polymer chains may also be crucial for this fluorescence quenching phenomenon by orienting the PA molecules in the polymer aggregates in an efficient way for the electron-transfer quenching process [84,85].

With regard to the superamplication quenching effect of the present fluorescent probes, the three-dimensional morphological structures of the polymer nanoaggregates and their AEE features may work collectively to make P**1**/**2**-I and P**1**/**2**-II highly sensitive to the analytes. Compared with the conventional solution-based sensors, the polymer nanoaggregates in the present sensing systems could bind with more quencher molecules due to the presence of numerous cavities in their three-dimensional morphological structures [68,70]. These three-dimensional cavities can provide additional pathways for the interchain diffusion of excitons and enable the entered quencher molecules like PA to efficiently interact with multiple TPE units in the polymer chains to quench the emission. Moreover, the distinct AEE feature of P**1**/**2**-I and P**1**/**2**-II endows their nanoaggregates with efficient fluorescence, which can also contribute to the high sensitivitiy [86]. Consequently, the quenching process of the nanoaggregates of these AEE-active polymers is more efficient than conventional solution-based sensors and is highly sensitive to the quencher concentration.

In addition to the “wet” method, we also explored the possibility of using the test strips of P**1**/**2**-I and P**1**/**2**-II for the PA detection. These polymer-coated strips were fabricated by simply dipping filter paper strips into the polymer solutions [80]. As shown in Figure S8, after solvent evaporation, the obtained test strips exhibit strong fluorescence under 365 nm UV irradiation from a hand-held UV lamp. When the test strips are partially dipped into the aqueous PA solution, the PA-covered parts become almost non-emissive due to the emission quenching by the PA molecules. This result indicates that the explosive detection using the AIE-active P**1**/**2**-I and P**1**/**2**-II can also be performed in the solid state to facilitate the real-world applications.

## 4. Conclusions

In summary, functional stereoregular polymers with 1,5-dihalo-(*Z*,*Z*)-1,4-pentadiene as dominated segments are prepared in this work through the metal-free multicomponent polymerizations of diyne, aldehyde and boron trihalide. The stereoselectivity of the polymerization was demonstrated to be highly dependent on the employed boron trihalide species as well as the reaction conditions. The polymerization of diyne, aldehyde and BCl_3_ that proceeds at 0 °C for 1 h followed by stirring at room temperature for another 1 h affords poly(dichloropentadiene)s with a predominant (*E*,*Z*)-configuration. When diyne and BCl_3_ are allowed to react at 45 °C for 1 h prior to further coupling with aldehyde at 0 °C and room temperature, a high molecular weight polymer (P**1**/**2**-I, *M*_w_ = 119,700) with the same composition but mainly (*Z*,*Z*)-configuration was obtained in a satisfactory yield. The content of (*Z*,*Z*)-isomeric unit of P**1**/**2**-I was determined to be up to 92%. Replacement of the mediator from BCl_3_ to BBr_3_ also results in polymers with a predominant (*Z*,*Z*)-1,4-pentadiene unit (P**1**/**2**-II) in a good yield of 79% with a high *M*_w_ of 63,100. All the obtained polymers possess good solubility and high thermal stability. The presence of numerous aromatic rings and halogen atoms in the polymer structures endows them with high refractive indices. Together with their good film-forming ability and low optical dispersion, the present polymers are promising to serve as coating materials in advanced photonic devices. The introduction of TPE moiety enables the polymers to show obvious AEE features. The fluorescence of the polymer nanoaggregates in aqueous media can be efficiently quenched by PA with a superamplification effect and a low detection limit, suggesting that they can be used for the sensitive detection of explosives.

## Figures and Tables

**Table 1:** BCl_3_-mediated polymerizations of **1** and **2** at different temperatures.

No.	*T* (°C)	Yield (%)	*M*_w_^b^	*M*_w_/*M*_n_^b^
1 ^a^	55	gel		
2 ^a^	45	68	119,700	3.9

^a^ Reactions were carried out under the following conditions: **1** and BCl_3_ (1.0 M in hexane) were reacted at *T* °C in DCM under nitrogen for 1 h. Monomer **2** was then added at 0 °C and the reaction mixture was kept at 0 °C for 1 h. After being warmed to room temperature, the reaction was continued for another 1 h. [**1**] = [**2**] = 0.2 M, [BCl_3_] =1.1 [**1**]; ^b^ Determined by GPC in THF on the basis of a linear polystyrene calibration.

**Table 2:** BBr_3_-mediated polymerizations of **1** and **2** under different conditions.

No.	*t*_1_ (h)	*t*_2_ (h)	Yield (%)	*M*_w_^b^	*M*_w_/*M*_n_^b^
1 ^a^	4	0	67	6900	2.3
2 ^b^	4	1	gel		
3 ^b^	2	1	79	63,100	4.3
4 ^b^	1	1	77	47,500	3.6

^a^ Reactions were carried out under the following conditions: **1**, **2** and BBr_3_ (1.0 M in DCM) were reacted in DCM under nitrogen at −40 °C for *t*_1_ h first. After being warmed to room temperature, the reaction was continued for another *t*_2_ h. [**1**] = [**2**] = 0.2 M, [BBr_3_] = 1.1 [**1**]; ^b^ Determined by GPC in THF on the basis of a linear polystyrene calibration.

## Supplementary Materials

The following are available online at http://www.mdpi.com/2073-4360/10/8/821/s1, Scheme S1: synthetic route of P**1**/**2**; Figure S1: absorption spectra of P**1**/**2**-I and P**1**/**2**-II in THF solutions; Figure S2: plot of fluorescence quantum yield of (a) P**1**/**2**-I and (b) P**1**/**2**-II versus water fraction in THF/water mixtures; Figure S3: particle size distributions of the nanoparticles of P**1**/**2**-I measured by dynamic light scattering; Figure S4: particle size distributions of the nanoparticles of P**1**/**2**-II measured by dynamic light scattering; Figure S5: fitting curve of P**1**/**2**-I for the calculation of the quenching constant; Figure S6: fitting curve of P**1**/**2**-II for the calculation of the quenching constant; Figure S7: normalized absorption spectrum of PA in water and PL spectra of P**1**/**2**-I and P**1**/**2**-II nanoaggregates in THF/water mixtures with 80% water fraction; Figure S8: fluorescence images of test strips coated with (a) P**1**/**2**-I and (b) P**1**/**2**-II before and after being partially dipped into an aqueous PA solution.
