# Supplementary material for: Functional Poly(dihalopentadiene)s: Stereoselective Synthesis, Aggregation-Enhanced Emission and Sensitive Detection of Explosives
Source: Polymers (Basel). 2018 Jul 25;10(8):821. doi: 10.3390/polym10080821 (PMC6403696; doi:10.3390/polym10080821)
Supplement: Supplementary file 1 [file polymers-10-00821-s001.pdf]

Electronic Supporting Information

Functional Poly(dihalopentadiene)s: Stereoselective Synthesis, Aggregation-Enhanced Emission and Sensitive Detection of Explosives

Ting Han,[1,2] Yun Zhang,[1,2] Benzhao He,[1,2] Jacky W. Y. Lam,[1,2,] and Ben Zhong Tang[1,2,3,*]*

[1]HKUST-Shenzhen Research Institute, No. 9 Yuexing 1st RD, South Area, Hi-tech Park, Nanshan, Shenzhen 518057, China

[2]Department of Chemistry, The Hong Kong Branch of Chinese National Engineering Research Center for Tissue Restoration and Reconstruction, Institute for Advanced Study, Department of Chemical and Biological Engineering and Division of Life Science, The Hong Kong University of Science and Technology, Clear Water Bay, Kowloon, Hong Kong, China

[3]NSFC Center for Luminescence from Molecular Aggregates, SCUT-HKUST Joint Research Institute, State Key Laboratory of Luminescent Materials and Devices, South China University of Technology, Guangzhou 510640, China

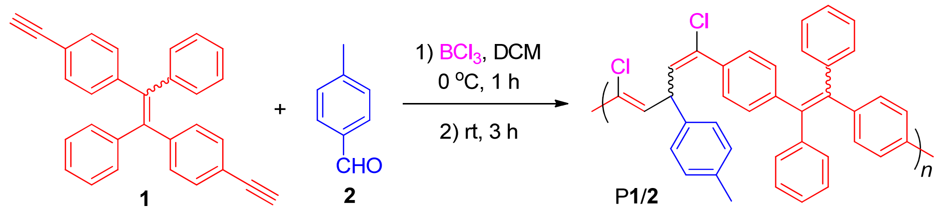

Scheme S1. Synthetic route of P**1**/**2**.

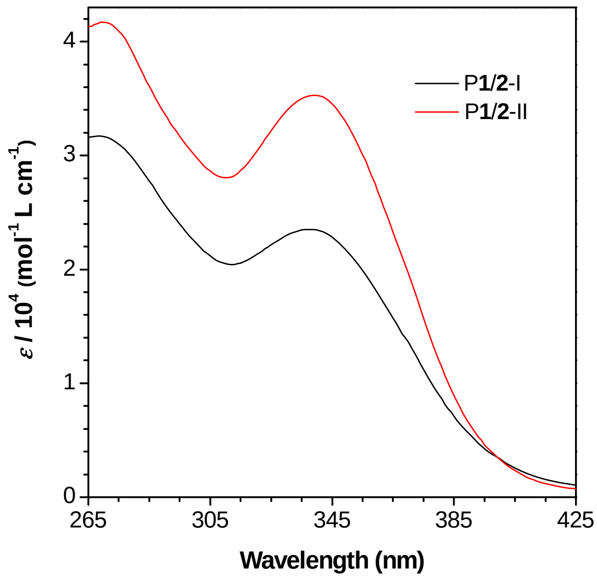

Figure S1. Absorption spectra of P**1**/**2**-I and P**1**/**2**-II in THF solutions. Solution concentration: 10 μM.

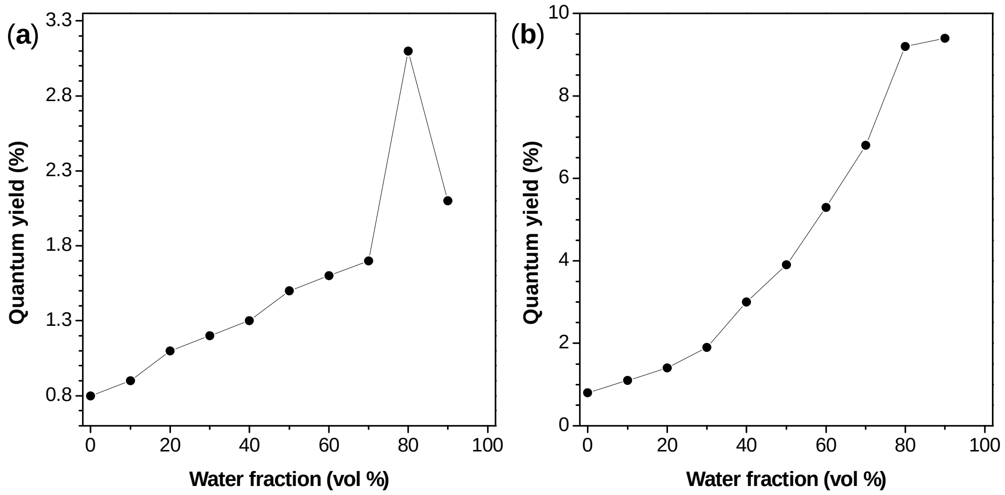

Figure S2. Plot of fluorescence quantum yield of (**a**) P**1**/**2**-I and (**b**) P**1**/**2**-II versus water fraction in THF/water mixtures. Solution concentration: 10 μM; excitation wavelength: 350 nm.

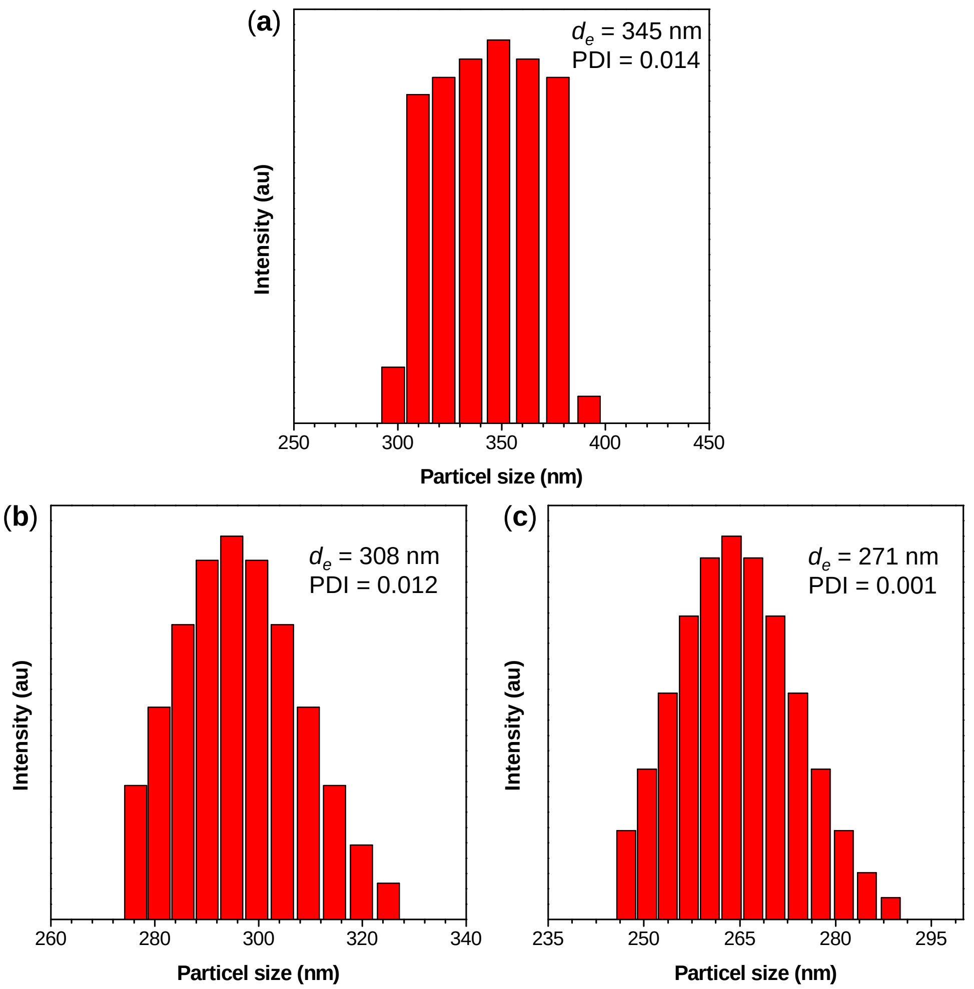

Figure S3. Particle size distributions of the nanoparticles of P**1**/**2**-I in THF/water mixtures with (**a**) 70%, (**b**) 80%, and (**c**) 90% water fraction measured by dynamic light scattering. Abbreviation: d_e = effective diameter, PDI = polydispersity index.

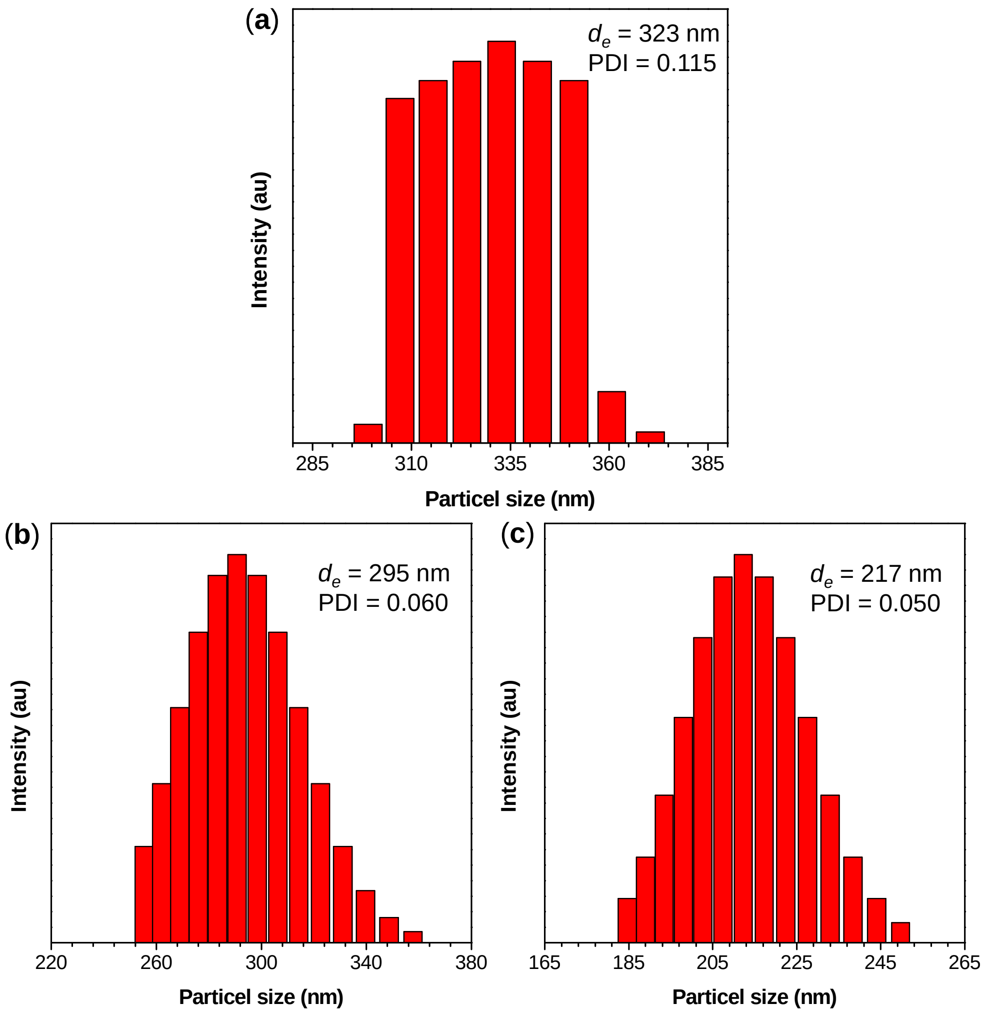

Figure S4. Particle size distributions of the nanoparticles of P**1**/**2**-II in THF/water mixtures with (**a**) 70%, (**b**) 80%, and (**c**) 90% water fraction measured by dynamic light scattering. Abbreviation: d_e = effective diameter, PDI = polydispersity index.

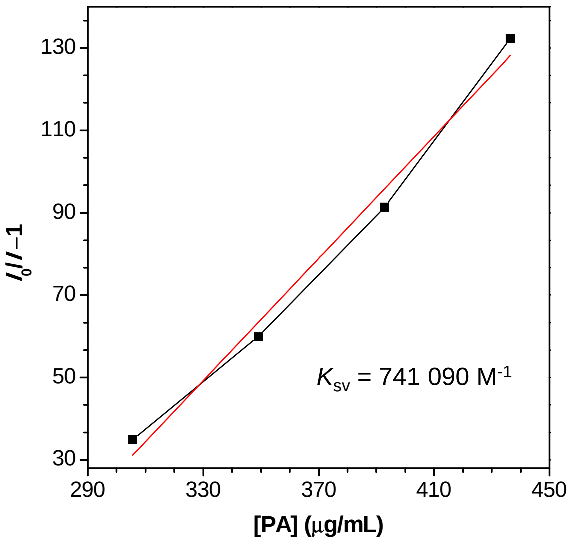

Figure S5. Fitting curve of P**1**/**2**-I for the calculation of the quenching constant.

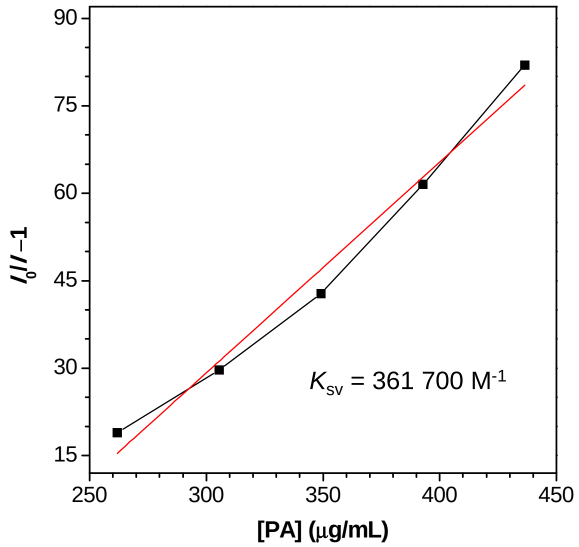

Figure S6. Fitting curve of P**1**/**2**-II for the calculation of the quenching constant.

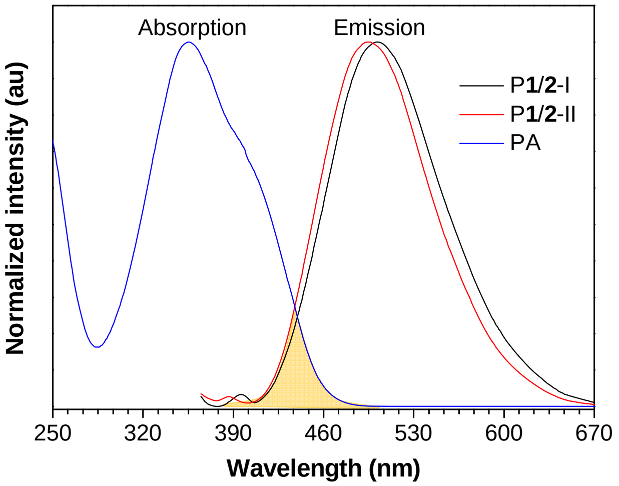

Figure S7. Normalized absorption spectrum of PA in water and PL spectra of P**1**/**2**-I and P**1**/**2**-II nanoaggregates in THF/water mixtures with 80% water fraction.

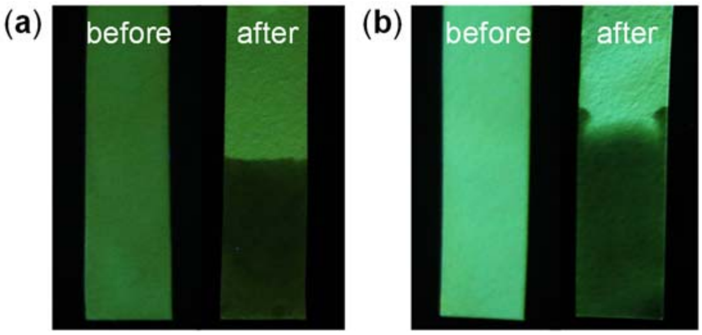

Figure S8. Fluorescence images of test strips coated with (**a**) P**1**/**2**-I and (**b**) P**1**/**2**-II before and after being partially dipped into an aqueous PA solution.
